# Report of trisomy 2q34-qter and monosomy 4q35.2-qter in a child with mild dysmorphic syndrome and karyotype 46,XY,der(4)t(2;4)(q34;q35.2)pat

**DOI:** 10.1186/s13039-020-00484-4

**Published:** 2020-05-19

**Authors:** Juan Pablo Meza-Espinoza, Enrique Sáinz González, Christian J. N. León-León, Eliakym Arámbula-Meraz, José Alfredo Contreras-Gutiérrez, Noemí García-Magallanes, Jesús Madueña-Molina, Fred Luque-Ortega, Salvador Cervín-Serrano, Verónica Judith Picos-Cárdenas

**Affiliations:** 1 Facultad de Medicina e Ingeniería en Sistemas Computacionales de Matamoros, Universidad Autónoma de Tamaulipas, Matamoros, Tamps., Mexico; 2 Servicio de Medicina Genética, Hospital General de Culiacán, Culiacán, Sin., Mexico; 3 Unidad Médica Familiar 11, Instituto Mexicano del Seguro Social (IMSS), Villa Juárez, Navolato, Sin., Mexico; 4 Laboratorio de Genética y Biología Molecular, Posgrado en Ciencias Biomédicas, Facultad de Ciencias Químico Biológicas, Universidad Autónoma de Sinaloa, Culiacán, Sin., Mexico; 5 Facultad de Medicina, Universidad Autónoma de Sinaloa, Culiacán, Sin., Mexico; 6 Laboratorio de Biomedicina y Biología Molecular, Unidad Académica de Ingeniería en Biotecnología, Universidad Politécnica de Sinaloa, Mazatlán, Sin., Mexico; 7 Facultad de Odontología, Universidad Autónoma de Sinaloa, Culiacán, Sin., Mexico; 8 Laboratorio de Genética, Facultad de Medicina, Universidad Autónoma de Sinaloa, Culiacán, Sin., Mexico

**Keywords:** Duplication 2q34-qter, Deletion 4q35.2-qter, der(4)t(2;4)(q34;q35.2), aCGH

## Abstract

**Background:**

Concomitant trisomy 2q3 and monosomy 4q3 have been rarely reported. Pure trisomy 2q3 has been associated with microcephaly, hypertelorism, low-set ears, micrognathia, visceral abnormalities, and growth retardation. Monosomy 4q3 includes a wide variety of dysmorphic features such an abnormal skull shape, hypertelorism, Pierre Robin sequence, short nose with abnormal bridge, fifth finger clinodactyly, congenital heart, and genitourinary defects, in addition to intellectual disability, developmental delay, and hypotonia, but more distal deletions involving 4q34-qter may result in milder phenotypes. Here, we present a child with a mild dysmorphic syndrome, resulted of a duplication 2q34-qter and a deletion 4q35.2-qter inherited of his father.

**Case presentation:**

We report a child, who at birth presented hypotonia, dysmorphism, and bilateral cryptorchidism. At 2 years and 9 month of age he showed brachycephaly, narrow forehead, bilateral frontoparietal hypertrichosis, down slanting palpebral fissures, sparse eyebrows, sparse short eyelashes, hypertelorism, depressed nasal root, broad nasal bridge, bulbous nasal tip, prominent colummela, broad nasal ala, smooth filtrum, high arched palate, thin upper lips, and ears rotated backwards. He also showed telethelia, hypertrichosis from dorsal to the sacral region, hands with clinodactyly and hypoplasia of the terminal phalanx of the fifth finger, and broad thumbs, broad first toes, and right cryptorchidism. A chromosomal study revealed a karyotype 46,XY,der(4)t(2;4)(q34;q35.2), while an array comparative genomic hybridization showed a 31.12 Mb duplication of the chromosome 2q34-q37.3 and a 1.49 Mb deletion in the chromosome 4q35.2.

**Conclusions:**

To our knowledge, only four families with translocation t(2;4) have been reported, two of them involving t(2q;4q), but the breakpoints involved in our patient have not been previously observed. The genomic imbalance in this patient was a duplication of 318 genes of the region 2q34-q37.3 and a deletion of 7 genes of 4q35.2. We discuss difficulty to assign specific congenital abnormalities to these duplicated/deleted regions and include some cases with terminal deletions of 4q with normal or just mildly detectable phenotypic effects.

## Background

A derivative chromosome results more commonly from reciprocal translocations, which could lead to gametes with some duplicated and other deleted chromosomal segments that could produce trisomies and monosomies at offspring [1]. Generally, unbalanced chromosomal abnormalities cause multiple malformations, growth delay, and mental retardation. Usually, affectation depends on chromosome and length of the implicated segment. Concomitant trisomy 2q3 and monosomy 4q3 have been sparsely reported [2, 3]. On a side, trisomy 2q3 has been associated with microcephaly, hypertelorism, low-set ears, micrognathia, visceral abnormalities, and growth retardation [3, 4]. It has been suggested that duplication 2q34-qter plays a key role in this phenotype [5]. Whereas monosomy 4q3, shows also clinical heterogeneity, which rely on the involved chromosomal region; a wide variety of dysmorphic features includes abnormal skull shape, hypertelorism, Pierre Robin sequence, short nose with abnormal bridge, abnormal fifth finger, congenital heart and genitourinary defects, intellectual disability, developmental delay, and hypotonia [6, 7]. However, more distal deletions that involve the 4q34-qter region may result in a milder phenotypic effect [8]. We report the case of a child with mild dysmorphic syndrome occasioned by trisomy 2q34-qter and monosomy 4q35.2-qter, consequence of a karyotype 46,XY,der(4)t(2;4)(q34;q35.2) inherited of his father.

## Case presentation

**Fig. 1:**
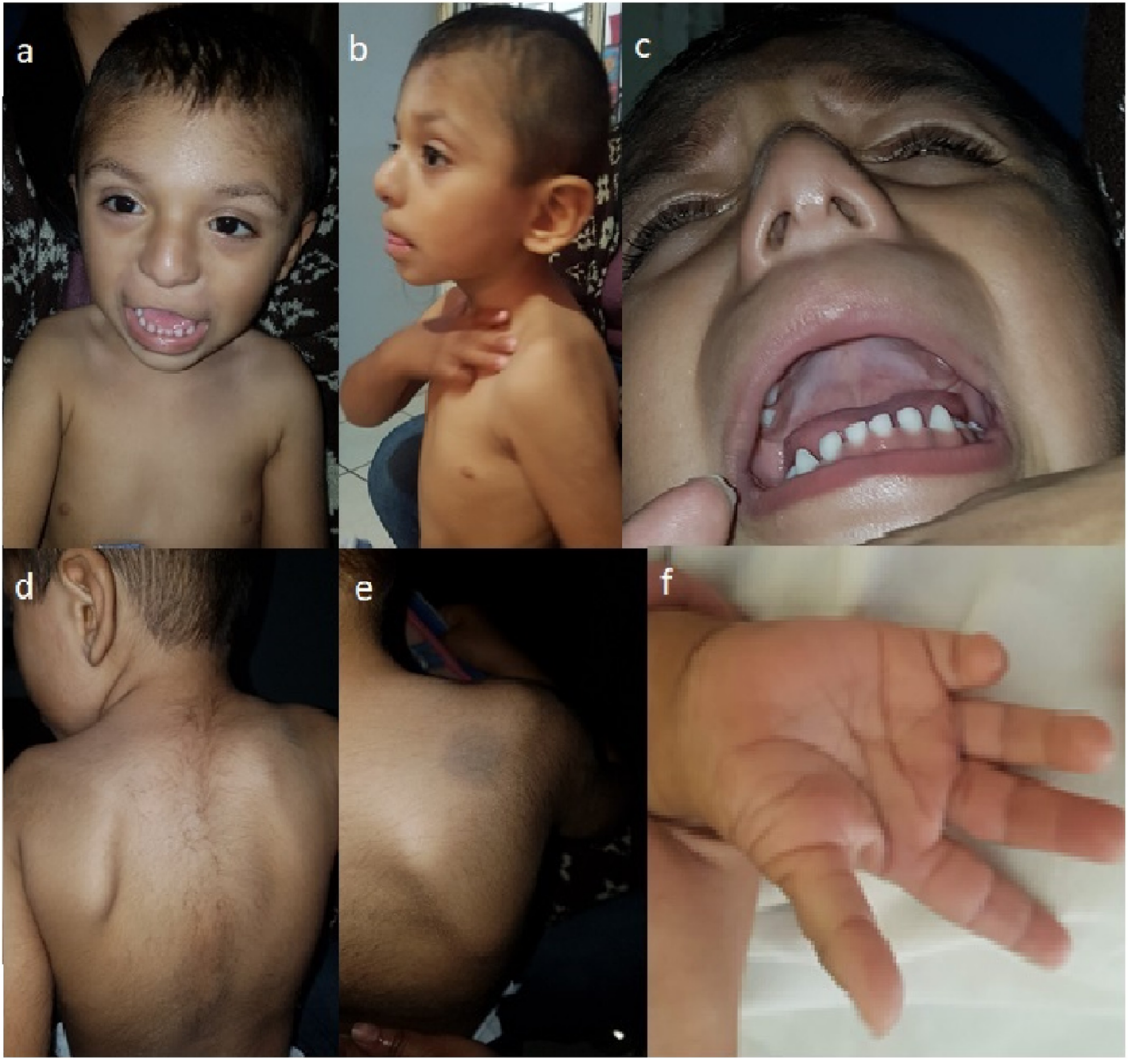
Clinical features of the proband. **a** Narrow forehead, bilateral frontoparietal hypertrichosis, hypertelorism, sparse eyebrows, down slanting palpebral fissures, sparse short eyelashes, smooth filtrum, thin upper lip, and telethelia. **b** Brachycephaly, depressed nasal root, broad nasal bridge, bulbous nasal tip, prominent colummela, broad nasal ala, and large ears rotated backwards. **c** High arched palate. **d** Hypertrichosis. **e** Hypermelanosis in the right scapular region. **f** Clinodactyly and hypoplasia of the terminal phalanx of the fifth finger, broad thumb, and deep creases on right hand

The proband is a 2 years 9 months old male, product of the first and at this time only pregnancy of healthy non-consanguineous young parents (mother was 24 years-old and the father 25). He was born by caesarean at 40 weeks of gestation due to cephalopelvic disproportion, with a weight of 3150 g (>30th centile, ~ 0.5 SDS), length of 50 cm (>50th centile, ~ 0 SDS), head circumference of 35 cm (>60th centile, ~ 0.4 SDS), thoracic circumference of 36 cm (>85th centile, ~ + 1 SDS), abdominal circumference of 30 cm, but with hypotonia, dysmorphism, bilateral cryptorchidism, and transient tachypnea. At present time, a physical examination revealed the next auxological parameters: height 86 cm (1.4th centile, ~ − 2.3 SDS), weight 12.4 kg (18.4th centile, ~ − 1 SDS), and head circumference 48 cm (20th centile, ~ − 0.9 SDS). His craniofacial features included brachycephaly, flat occiput, punctiform posterior fontanel, bilateral frontoparietal hypertrichosis, short neck, low set ears rotated backwards, downslanted palpebral fissures, hypertelorism, thin upper lip, upwards labial commisures, depressed nasal root, broad nasal bridge, bulbous nasal tip, broad nasal ala, prominent colummela and smooth filtrum (Fig. 1a and b). Other phenotypic abnormalities included widely spaced nipples (Fig. 1a); high arched palate (Fig. 1c); hypertrichosis from dorsal to the sacral region (Fig. 1d); dermal hypermelanosis in the right scapular region (Fig. 1e); Clinodactyly and hypoplasia of the terminal phalanx of the fifth finger in both hands (Fig. 1f); broad first toes with dysplastic nails and deep creases on right foot plant; hyperplastic scrotum, right cryptorchidism and hypoplastic left testicle. Moreover, a murmur in aortic focus 2–4/6 was detected (but no echocardiographic evidence of heart anomalies). The family antecedents did not indicate any significant information and the mother denied complications during pregnancy or exposure to teratogens. The proband presents delayed psychomotor development: he was able to sit up at 12 months of age and to say monosyllables at 18 months of age, and he walked at 22 months of age. Currently the patient has no serious complications and he shows good reflexes and muscle tones, but he has a mild intellectual disability and lacks of speech.

## Results

**Fig. 2:**
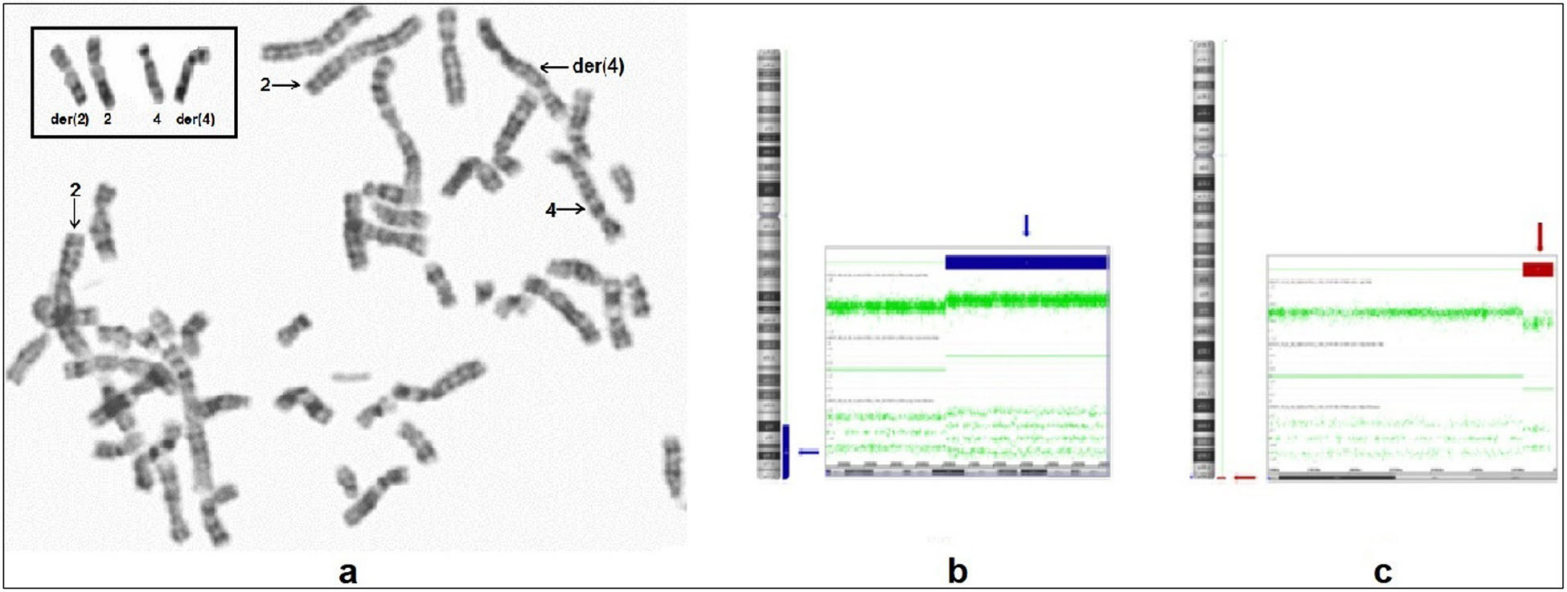
Results of the chromosomal and aCGH analysis. **a** GTG-banded metaphase obtained of the proband. The arrows indicate the chromosomes involved in the imbalance. Moreover, in the box upper left corner are added the derivatives chromosomes obtained from the karyotype t(2;4)(q34;q35.2) of the father. **b** Image of the aCGH showing a 31.12 Mb duplication of the chromosome 2q34-q37.3 (210,718,096-241,840,106); the blue arrows and bars indicate the duplicated region. **c** aCGH plot showing a 1.49 Mb deletion of the chromosome 4q35.2 (188,545,291-190,036,305); the red arrows and bars point the deleted region

A chromosomal analysis from peripheral blood cells in the proband revealed a chromosome derivative 4 with an apparent addition at 4q35. However, karyotyping in the parents showed that the father was a carrier of a balanced reciprocal translocation 46,XY,t(2;4)(q34;q35.2) and consequently the karyotype in the child was assigned as 46,XY,der(4)t(2;4)(q34;q35.2)pat (Fig. 2a). The karyotype of both paternal grandparents was normal. Moreover, to determinate the genomic imbalance, array comparative genomic hybridization (aCGH) on the proband was performed through CytoScan™ Technology (Thermo Fisher Scientific Inc) according to recommendations of the supplier. Data were analyzed with the ChAS 4.0 software. Interpretation of results was realized through the following databases: DGV, Cytogenomics Array Group CNV Database, Ensembl Resources, OMIM, UCSC, ClinGen, and ClinVar. This analysis showed a duplication of 31.12 megabases (Mb) of the chromosome 2q34-q37.3: arr[GRCh38] 2q34q37.3(210,718,096-241,840,106)×3 containing 318 genes (Fig. 2b), and a deletion of 1.49 Mb in the chromosome 4: arr[GRCh38] 4q35.2(188,545,291-190,036,305)×1 spanning 7 genes (Fig. 2c).

## Discussion and conclusions

The karyotype results and aCGH findings of our patient were 46,XY,der(4)t(2;4)(q34;q35.2)pat and arr2q34q37.3(210,718,096-241,840,106)x3pat,arr4q35.2(188,545,291-190,036,305)x1pat, respectively. The genomic imbalance was a 31.12 Mb duplication of the chromosome 2q34-q37.3, encompassing 318 genes, and a 1.49 Mb deletion in the chromosome 4, spanning 7 genes (*LINC01060, LINC02508, LINC01262, FRG1-DT, LINC01596, FRG1, and FRG2*). To our understanding, only four families with translocation t(2;4) have been reported [2, 3, 9, 10], two of them involving t(2q;4q), with three surviving patients inheriting the chromosome der(4) [2, 3] [Table 1]. However, the breakpoints implicated in our patient have not been previously observed. In the case described by Rashidi-Nezhad et al. [2] the imbalance was a 32.9 Mb duplication spanning 2q34-q37.3 and a 13.5 Mb deletion of 4q34.2-q35.2; while in the subjects reported by Ronzoni et al. [3] the imbalance corresponds to a 26 Mb duplication of 2q35-q37.3 and a 6.3 Mb deletion of 4q35.1-q35.2. Our patient shares a similar phenotype to the individual reported by Rashidi-Nezhad et al. [2], as hypotonia, flat occipital, broad nasal bridge, high arched palate, thin upper lip, bilateral clinodactyly of the fifth finger, right cryptorchidism, and hypoplastic left testicle. While, the male described by Ronzoni et al. [3], shares more phenotypic similarity than the female, principally brachycephaly, high arched palate, and nail dysplasia of feet. Our patient presented some comparable features with cases of pure trisomy 2q35-qter, as low set ears, prominent nasal tip, thin upper lip, fifth finger clinodactyly, cryptorchidism, and normal body measurements at birth [11, 12]. But, severity of the phenotype is major in proximal duplications to 2q33 [4, 13]. Like our case, many subjects with trisomy 2q3 result from parental balanced chromosomal abnormalities and consequently they have also monosomy of another chromosomal segment; therefore, it has been difficult to assign specific congenital malformations to duplication of this region. Still, Rashidi-Nezhad et al. [2], proposed that 2q36.2-qter duplication and 2q34 proximal duplication (chr2: 209,778,861) could be critical for fifth finger clinodactyly and for congenital heart defects, respectively. Accordingly, our patient did not show cardiac anomalies. On the other hand, apart our case, seven of eight male patients with trisomy overlapping the 2q35-q37 region, four of them with 2q pure trisomy, had testicle abnormalities [2, 3, 11, 12, 14–17], while, three of seven comparable females had genitourinary alterations [2, 4, 13, 18–21]. On this basis, we hypothesize that duplication of this region could be directly related with such as defects, these being more penetrant in males. It is remarkable that even though our patient had an excess of 318 genes of the segment 2q34-q37.3, he did not show serious malformations. Regarding monosomy 4q35-qter, Vona et al. [22] described a child that had a deletion 4q35.1-q35.2, spanning 6.9 Mb (chr4:184,046,156-190,901,117), who was found with congenital hearing impairment and a moderate clinical phenotype. Meanwhile, Descartes et al. [23] found a terminal deletion 4q34.2 in patients with growth retardation, intellectual disability, and craniofacial alterations. Tsai et al. [24] reported a case with deletion 4q34.2-qter and cardiac defects, cleft palate, learning difficulties, and right fifth finger anomalies. Rossi et al. [25] described a female with a deletion 4q34.1-q35.2 extending 16.43 Mb (chr4: 174,685,919-191,121,195) that presented Pierre Robin sequence, cardiac abnormalities, and learning disabilities. All these cases share some features with our patient, principally intellectual disability. Strehle et al. [7] based on observation of a patient who showed almost all the clinical features of 4q deletion syndrome had a loss approximately 465 kb at 4q35.1 (chr4: 186,770,069-187,234,800) suggested that the deletion of this region is critical for expression of that syndrome. However, some cases with deletions in 4q35 and only intellectual or learning disability have been detected [25–28]. Even more, individuals with no detectable phenotypic effects and terminal chromosomal deletions in 4q have also been described. First, a male with a deletion 4q34.2-qter, who transmitted it to his daughter, but she suffered severe atrial septal defect and died perinatally [29]; also, a male with a subtelomeric deletion 4q, who had two children with developmental delay and mental retardation [30]. A female with a deletion 4q35, spanning 1.15–1.3 Mb, who had two mentally retarded children [31], a female with an interstitial deletion in 4q34.1-q34.3, spanning at least 9.3 Mb, but she had three successive miscarriages [32], and a female and her two daughters with an interstitial deletion 4q35.1–35.2 encompassing 5.75 Mb (chr4: 184,717,878-190,469,337) [33]. The reason why such deletions produce clinical phenotype in some individuals but not in others is unknown; however, modifier genetic variants and/or epigenetic changes may be involved. Therefore, it is difficult to realize precise genotype-phenotype correlations. Yet, we believe that the phenotypical abnormalities found in our patient are consequence of the duplication 2q34-qter, since there is excess of 318 genes of this region, and deletion of “only” seven genes of 4q35.2. Moreover, the most common features of patients with 2q35-2q37.3 duplication are found in this case. Finally, is probable that this translocation had been originated during gametogenesis of any of the paternal grandparents. Once again, it is demonstrated that parental karyotyping is important to determine origin of chromosomal abnormalities and involved breakpoints, moreover for genetic counselling. However, aCGH is necessary to determine genomic imbalance and gradually delineate genotype-phenotype correlations, since clinical manifestations are, usually, genes dosage-dependent.

**Table 1:** Clinical findings in subjects with chromosome der(4) involving t(2;4)(q3;q3)

Reference	Rashidi-Nezhad et al., *2012* [2]	Ronzoni et al., *2015* [3]	Ronzoni et al., *2015* [3]	The present case
**Karyotype**	46,XY,der(4)t(2;4)(q34;q34)mat	46,XY,der(4),t(2;4)(q35;q35)pat	46,XX,der(4),t(2;4)(q35;q35)mat	46,XY,der(4)t(2;4)(q34;q35.2)pat
**array comparative genomic hybridization**	arr2q34-q37.3(209,778,861-242,706,291) x 3mat,arr4q34.2q35.2(177,717,642-191,220,565)x1mat.	arr2q35-q37.3(216,850,125-243,007,359) x3pat,4q35.1-q35.2(184,605,094–190,896,674)x1pat.	arr2q35-q37.3(216,850,125-243,007,359)x3mat,arr4q35.1-q35.2(184,605,094-190,896,674)x1mat.	arr2q34-q37.3(210,718,096-241,840,106)x3pat,arr4q35.2(188,545,291-190,036,305)x1pat.
**Trisomy**	2q34-q37.3	2q35-q37.3	2q35-q37.3	2q34-q37.3
**Monosomy**	4q34.2-q35.2	4q35.1-q35.2	4q35.1-q35.2	4q35.2
**Normal birth measurements**	+	+	–	+
**Hypotony at birth**	+	+	–	+
**Microcephaly**	–	–	–	–
**Brachycephaly**	+	+	–	+
**Prominent forehead**	+	–	–	–
**Hypertelorism**	–	–	–	+
**Down slanting palpebral fissures**	–	–	–	+
**Ears abnormalities**	+	+	+	+
**Sensorineural hearing loss**	+	+	+	–
**Depressed nasal root**	–	–	–	+
**Nasal anomalies**	+	+	–	+
**Long philtrum**	+	–	–	–
**Thin upper lip**	+	–	–	+
**High palate**	+	+	–	+
**Digital anomalies**	+	+	+	+
**Heart Malformations**	–	–	–	–
**Genitourinary anomalies**	+	+	+	+
**Growth retardation**	–	–	–	+
**Psychomotor delay**	+	+	+	+
**Intellectual disability**	+	+	+	+

## Data Availability

Data generated or analyzed during this study are included in this published article.
